# Primary Erythromelalgia: Historical Perspective and Current Update

**DOI:** 10.7759/cureus.78576

**Published:** 2025-02-05

**Authors:** Allison C Eaton, Harvey N Mayrovitz

**Affiliations:** 1 Medical School, Nova Southeastern University Dr. Kiran C. Patel College of Osteopathic Medicine, Davie, USA; 2 Medical Education, Nova Southeastern University Dr. Kiran C. Patel College of Allopathic Medicine, Davie, USA

**Keywords:** erythermalgia, erythromelalgia, nav1.7, neuropathic pain, scn9a, skin erythema, sodium channelopathy

## Abstract

Erythromelalgia is a condition characterized by intense burning pain, redness, and heat in the extremities that has garnered increasing attention in recent years. This literature review provides a comprehensive historical perspective and current update on primary erythromelalgia or PEM, categorizing and tracing the clinical knowledge of the condition and identifying key milestones of historical research. In a sequential fashion, the review explores the evolution of understanding of PEM, starting from its initial descriptions in the medical literature to the present day. Early case reports and pivotal studies that contributed to recognizing and characterizing the disorder are examined. Important discoveries, diagnostic criteria, and therapeutic approaches that have shaped the management of erythromelalgia over time are highlighted. Additionally, the impact of genetic studies and molecular investigations on current understanding of PEM is discussed. Identifying mutations in the SCN9A gene is emphasized as a significant breakthrough, shedding light on the role of sodium channels in the disorder's pathogenesis. Overall, this review consolidates the wealth of clinical knowledge and research milestones related to PEM. Integrating historical research milestones offers a comprehensive overview of the condition, from early descriptions to the current state of knowledge. This knowledge serves as a foundation for further research and can assist in improving diagnosis and management strategies for individuals with PEM.

## Introduction and background

Erythromelalgia is a rare and sometimes debilitating disorder characterized by episodes of intense burning pain, redness, and heat in the upper and lower distal extremities [1-3]. Though classically presenting unilaterally in the hands and feet, it can also manifest bilaterally. In rare cases, it may present on the face or ear [4]. It affects individuals of various age groups and can significantly impair their quality of life [5-7]. This paper presents a comprehensive narrative review of primary erythromelalgia (PEM), focusing on the historical milestones and current understanding of the disease. Though similar in symptomatology, PEM is to be distinguished from type 2 erythromelalgia that is secondary to myeloproliferative disorders such as polycythemia vera [3,5,8,9].

Epidemiologically, erythromelalgia has a low prevalence, estimated to affect less than two persons per 100,000 population [1,7]. However, the epidemiological data on erythromelalgia is limited, partly due to its rare nature and partly due to its potential underdiagnosis. The disorder is considered sporadic in most cases, although familial forms have been reported, suggestive of an underlying genetic component [3,9].

Throughout the literature, the term "erythermalgia" has sometimes been used interchangeably with "erythromelalgia" to describe this condition. However, it is worth noting that there has been a growing consensus among experts to move on from this historical term that has fallen out of common use. Therefore, in this paper, the term "erythromelalgia" will be used exclusively to refer to the disorder under investigation in order to maintain clarity and consistency with current medical literature.

This research aims to categorize and trace the clinical knowledge of PEM, shedding light on its historical progression. By reviewing significant research milestones, we aim to comprehensively understand the disorder from early descriptions to the present day. It is hoped that this will enable researchers, clinicians, and patients to gain insights into the advances made in diagnosing and managing PEM. Additionally, this review will explore the impact of genetic studies and molecular investigations on our current understanding of PEM. Identifying mutations in the SCN9A gene, encoding a sodium channel critical for pain perception, has emerged as a significant breakthrough, highlighting the underlying mechanisms of the disorder.

## Review

Methods

Electronic databases, including PubMed, Web of Science, ProQuest, and Google Scholar, were utilized to identify and review the literature. Search terms included "erythromelalgia," "erythermalgia," "SCN9A," and “sodium channelopathy.” Advanced search options (AND, OR) were used to combine search terms. Reference sections of relevant articles were reviewed to further establish the knowledge production trajectory. The inclusion criteria were as follows: publication in a scientific journal, texts published in English, studies focusing on either clinical or scientific aspects of erythromelalgia, and studies including a qualitative description of erythromelalgia pathology. As an analytic technique, thematic analysis was employed to identify critical moments in the history of knowledge production around erythromelalgia. Limitations include scarcity of erythromelalgia research and inconsistency in nomenclature utilized in some reviewed studies.

Red, hot, and painful: early descriptions of a mysterious disease

**Figure 1:**
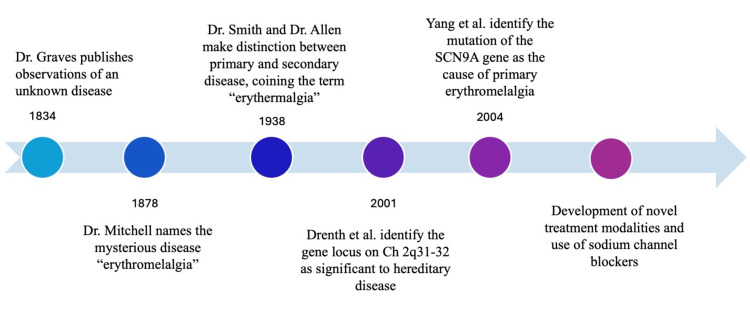
Milestone timeline The timeline features the most notable historical developments traced to our current understanding of PEM: Graves, 1834 [10]; Mitchell, 1878 [6]; Smith and Allen, 1938 [5]; Drenth et al., 2001 [11]; Yang et al., 2004 [9]. This is an original figure courtesy of the authors.

In the early literature on erythromelalgia, physician Silas Weir Mitchell is often recognized as the first to publish on the disease (Figure 1). His 1878 article consists of case studies of erythromelalgia and other related diseases, with the intention of distinguishing erythromelalgia from its counterparts. In a footnote on the first page, Mitchell coined the term "erythromelalgia" to describe the disease characterized by "throbbing, aching, [and] burning" sensations [6]. Derived from Greek roots, the term combines erythros (red), melos (limb or "a member"), and algos (pain). Mitchell referred to it as a "foot and hand disorder." These descriptions provided the foundation for understanding the unique pain and vascular symptoms associated with the disease.

Approximately 60 years later, physicians Lucian A. Smith and Edgar V. Allen made a significant contribution to the erythromelalgia literature with their 1938 paper [5]. They commenced their work with a critique of Mitchell's terminology, noting that it was incomplete as it failed to incorporate a crucial symptom: heat. Prior to their publication in 1933, another physician Thomas Lewis had suggested that the term "erythromelalgia" lacked a precise definition and proposed retiring it as a medical term [8,12]. However, instead of abandoning the term entirely, Smith and Allen proposed augmenting it by incorporating the root word "therm" to convey the element of heat explicitly. They suggested that the condition be called "erythermalgia." Despite these debates on nomenclature, Mitchell's original term, "erythromelalgia," gained widespread acceptance as the official designation and, as noted earlier, is the term used herein.

The early literature on erythromelalgia holds significance not only in terms of etymology but also for documenting the initial clinical descriptions of the disease and early attempts at treatment. Although scientific understanding of erythromelalgia has advanced significantly since then, the vivid qualitative descriptions found in early case studies remain valuable as clinical examples of erythromelalgia's presentation. Physicians observed and documented the distinct triad of symptoms and triggers for flare-ups associated with this condition. An excerpt from a case study in Smith and Allens’ 1938 article [5] is a quintessential example of such early qualitative descriptions. Referring to a 30-year-old immigrant woman from Poland, the authors wrote:

“She came to the United States in April, 1936, at which time the typical distress appeared. She described it as a severe burning and pricking sensation which involved both feet. The distress was made worse by wearing shoes, hot weather, walking, standing or sitting, and she had noticed that it occurred after a warm bath. She had obtained relief by bathing or swimming in cold water, by walking on cold floors, and by elevating her feet. When the distress was present, her feet felt swollen, appeared puffy and red, and felt quite hot to the touch. She slept with few covers and often uncovered her feet, even in cold weather. There was always a definite relation between the burning distress and the objective temperature of the feet. She found she could walk four or five blocks in cold weather, but only one block in warm weather, before the burning distress appeared.” 

Other case studies in the above-cited authors’ article include a foreman in a goldmine who experienced symptoms “precipitated and aggravated by work, walking, or climbing,” a woman whose symptoms were triggered by riding in hot trains and automobiles, a woman who experienced burning in her hands and feet brought on by factory work capping bottles and using an electric sewing machine, and a man who experienced burning pain in his right foot that was so severe he had to walk with a cane. Mitchell’s landmark 1878 paper [6] also consists almost solely of case studies. One of these cases features a sailor in the U.S. Navy that Mitchell describes as follows:

“The disease progressed rapidly, and when I saw the man, in June, his condition was no less strange than pitiable. He was a well-made, vigorous person, of rather ruddy complexion…He told me that he had pain in the feet whenever he attempted to talk, but that while at rest in bed he was perfectly comfortable. The case, as he spoke of it, was to me so novel that I somewhat mistrusted his statement, and, therefore, directed him to walk up and down the ward and about the groups until I sent for him, which I did when at the close of an hour my visit was over. He made his appearance in the ward, walking with the step of a man whose feet were tender. On examining his extremities I found them both swollen. They scarcely pitted on pressure, but were purple with congestion; the veins were everywhere singularly enlarged, and the arteries were throbbing visibly. The whole foot was said to be aching and burning, but above the ankles there was neither swelling, pain, nor flushing.”

**Figure 2:**
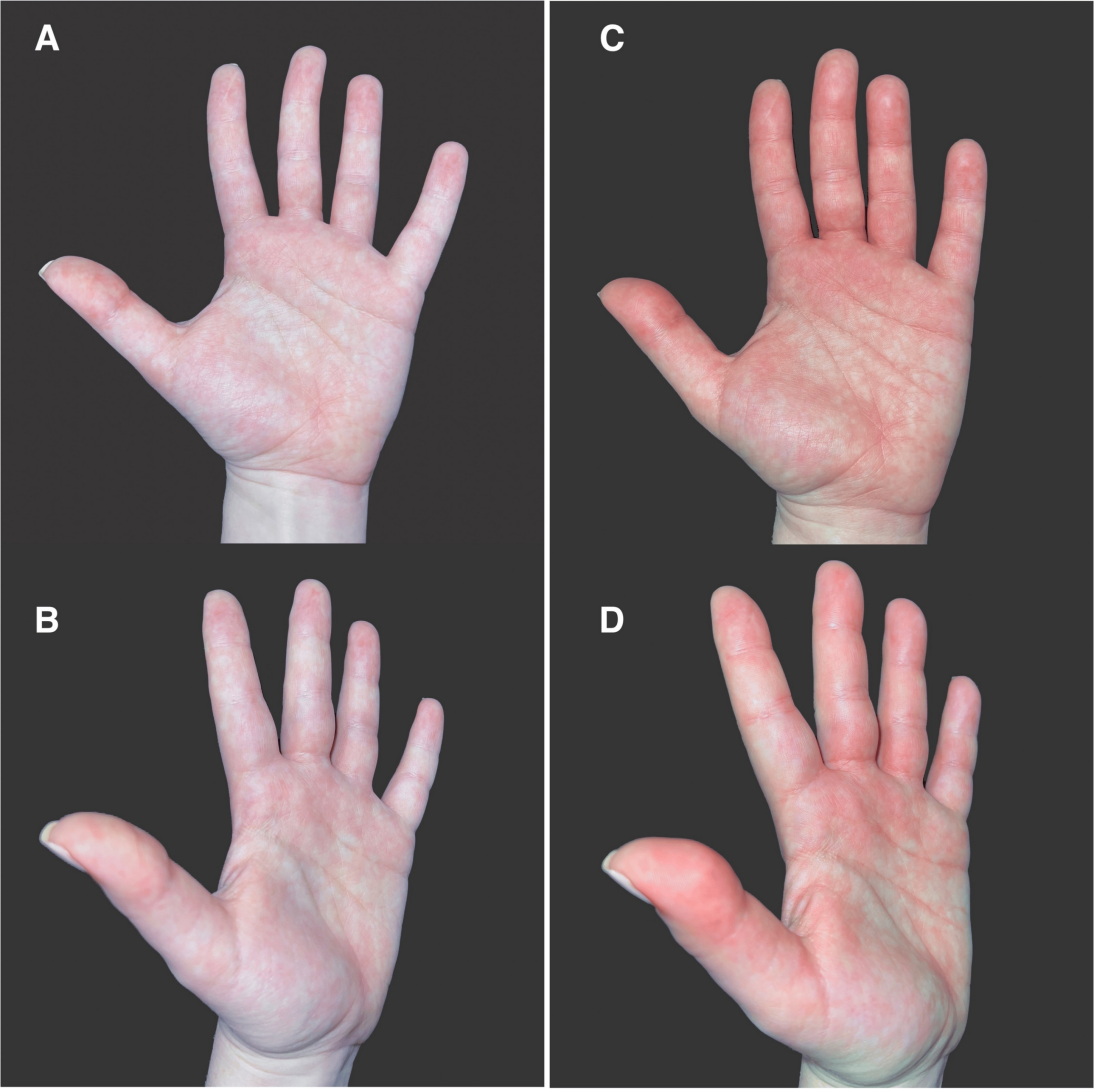
Example of a primary erythromelalgia flare Images (A and B) show two views of the left hand before the attack, and images (C and D) show two views of the left hand during the acute flare approximately 15 minutes later. This is an original figure courtesy of the authors.

To provide a current visualization of the condition, Figure 2 depicts events associated with the recent triggering and outcome of one of the authors who suffers from the condition. The subject has been clinically diagnosed with PEM, which has been present since early childhood. The images were taken before and after the flare, resulting from 15 minutes of light cardio exercise on an indoor treadmill, with the left hand kept in a dependent position. There is notable patchy erythema with mottling and swelling of the thumb and digits. There is an associated dissipation of heat and characteristic itch in the affected areas. The reaction is contained to the volar surface only.

Along with descriptions of symptoms, the early literature also documents treatment efforts. Early treatments for erythromelalgia were experimental. Sometimes, the physician or the patient themselves found helpful palliative forms of care. Other times, treatment efforts involved extreme procedures driven by a desire to alleviate the severe pain and discomfort. Many sources detail ice-bath immersion as a common treatment for erythromelalgia, as the intense cold temporarily relieved the burning sensation [2,3,6,8,13,14]. However, sometimes, this treatment resulted in frostbite or ulcerous “immersion foot” [14]. In addition, amputation was considered as a drastic measure to alleviate symptoms. In 1903, physician HB Shaw documented the case of a patient whose “pain was so severe and the affected extremity so useless that amputation was performed” [15]. The rationale behind amputation was based on the belief that removal of the affected limb would eradicate the pain and vascular disturbances. In another case, Shaw, referring to the practice of making incisions to treat erythromelalgia swelling, writes: “Incisions over such swelling, even down to the bone, have proved useless” [15]. Babb et al. wrote of other early treatment attempts performed on erythromelalgia patients: “Patients with severe [erythromelalgia] not responsive to conservative measures have been subjected to lumbar ganglionectomy or to blocking, crushing, or dividing of the peripheral nerves” [8]. The above interventions were not widely adopted due in part to their resulting complications. In the available cases, it is implied that these invasive procedures did not improve individuals’ quality of life or reduce the recurrence of flare-ups.

Along with the above-mentioned interventions, physicians in the late 1800s and early 1900s made sound recommendations that were simple, doable, and proved helpful to patients. Many such recommendations were common sense treatments that patients sometimes instinctively adopted on their own. These forms of palliative care are evidenced in the case studies of which the early literature is largely composed. Such treatments include avoiding warm temperatures, cooling the extremities with a fan or ice, keeping minimal coverage with blankets at night, avoiding exercise and prolonged dependent state, avoiding wearing full coverage shoes or socks, and elevating the affected extremity [5,6,8,16].

Pharmacological approaches also emerged during this period as attempts to manage symptoms of erythromelalgia. Treatment was often based on anecdotal evidence, and results had varying efficacy. Medications such as salicylates, which possess anti-inflammatory and analgesic properties, were employed in early treatment efforts [13]. While these drugs provided temporary relief for some individuals, they often fell short of providing consistent relief. In addition to the use of salicylates, there are several single case reports that indicate the experimental use of numerous medications such as digitalis, iron tincture, bromides, ergot, galvanism, liniment, quinine, colchicum root wine, camphor, hemlock extract, opium extract, and application of leeches [3,6,13]. It is unclear whether any therapeutic value was discovered in these early experimental treatment attempts. It is essential to recognize that the early treatments and interventions reflect the limited and inchoate understanding of the underlying mechanisms and pathophysiology of erythromelalgia in the early 20th century. The pioneering physicians of this era worked with the knowledge and resources available to them, and their efforts paved the way for further advancements in understanding and managing the condition.

Theories on the pathogenesis 

As previously outlined, in the late 19th century, medical and scientific publications on this disease were mostly descriptive case reports of limited value outside of outlining symptomatology and palliative care strategies. Early investigations into pathology were marked by a spectrum of theories rooted in clinical observations and evolving medical knowledge, which sought to elucidate the origins of erythromelalgia's distinctive symptoms. Ranging from vascular abnormalities to neuropathic hypotheses and considerations of platelet dysfunction, these pre-genetic theories reflect the evolving landscape of the early 20th-century scientific inquiry into erythromelalgia's complex pathophysiology. In this section, we provide an overview of the evolution of historical theories that once framed our understanding of erythromelalgia and paved the way for the genetic breakthroughs that would eventually reshape our comprehension of this mysterious condition.

Blood Flow Hypothesis

An essential development in the origins of PEM comes from investigations examining microcirculation and blood flow dynamics in affected areas. Studies focusing on individuals with erythromelalgia have revealed significant endothelial cell swelling as a distinctive pathological feature [17]. This swelling, along with structural alterations observed in cutaneous microvessels of the foot, potentially contributes to an imbalance in blood flow regulation. Such irregularities disrupt the typical control of blood vessel dilation and constriction responses, leading to the enhanced response of cutaneous microvessels to stimuli [18]. Some microvascular theories even go so far as to assert that PEM might be considered a condition caused by a common microvascular response, specifically microvascular arteriovenous shunting, rather than being viewed as a distinct disease entity [19]. Additionally, persistent skin vascular abnormalities such as cyanotic extremities and Raynaud’s phenomenon observed between flares suggest a sustained baseline vascular instability in erythromelalgia [20]. As theorized by Mørk et al., there is an increased action of thermoregulatory arteriovenous shunting during attacks of PEM [21]. Compensatory arteriolar dilatation in hypoxic regions, such as the plantar area, escalates skin perfusion and temperature. However, an existent imbalanced flow distribution away from hypoxic areas and toward anatomical arteriovenous shunts, combined with now heightened metabolic demands, perpetuates hypoxia, contributing to the cyclic nature of erythromelalgia pathogenesis [21]. These anomalies were theorized to contribute to the hallmark symptoms experienced during erythromelalgia episodes, including skin redness, heightened skin temperature, and the localized nature of symptoms.

Neuropathic Hypothesis

The exploration of erythromelalgia's etiology significantly revolved around neuropathic theories. The neuropathic theory evolved in part based on the fact that many patients presented with a distal small fiber neuropathy featuring selective involvement of cutaneous sympathetic fibers, implicated as a key factor influencing the condition's manifestation [22]. This neuropathic perspective highlighted the crucial role of altered peripheral nerve function and signaling pathways in the onset of PEM. Specifically, research findings suggested that postganglionic sympathetic dysfunction and denervation hypersensitivity might play a role in PEM, while local neurogenic function and endothelial function seemed unaffected [23,24]. Notably, at that stage of research preceding genetic studies, the distinction between whether the neuropathy associated with erythromelalgia represented a primary or secondary disorder remained unclear [23]. This intricate relationship between neural abnormalities and their impact on vascular dynamics underscored the complexity of the neuropathic mechanisms contributing to PEM, necessitating further thorough investigations into these specific pathways.

Combining Neuropathic and Vascular Theories

Erythromelalgia's historical understanding was shaped by two prevailing and interconnected theories - perspectives concentrating on neuropathic or microcirculatory pathological origin. These concurrent viewpoints delved into the intricate interplay between neural and vascular mechanisms, characterizing the comprehension of the condition. However, pivotal genetic discoveries in the late 20th century would prove to unveil underlying genetic aspects. This redirection from dominant vascular and neuropathic theories to emerging genetic paradigms centered around ion channel dysfunction propelled advancements in research, signifying a transformative phase in exploring erythromelalgia's complex pathophysiology.

Genetic advancement and current therapies

Genetic research has been instrumental in advancing our understanding of PEM since the early 2000s. In 2001, the identification of the locus of the unknown PEM susceptibility gene on chromosome 2q31-32 by Drenth et al. represented the first concrete evidence supporting the theory of a genetic component in erythromelalgia [11]. This discovery laid the groundwork for subsequent genetic studies and highlighted the importance of genetic factors in the development of the condition. Building upon this foundation, Yang et al. made a significant breakthrough in 2004, identifying the gene responsible for PEM as SCN9A [9]. This gene encodes the NaV1.7 voltage-gated sodium channel alpha subunit, predominantly expressed in nociceptive neurons localized on the dorsal root ganglia, where it plays an integral role in neuronal excitability and pain signaling [25,26]. More than 20 gain-of-function mutations of SCN9A have since been identified in PEM cases, with inheritance patterns observed in an autosomal dominant manner or sporadically [2,9,26,27]. These mutations have been shown to induce a hyperpolarizing shift in activation and slow deactivation, thereby amplifying the channel response to small depolarizing stimuli [28]. This heightened excitability in affected cells contributes to aberrant neuronal signaling, manifesting in the hallmark symptoms of erythromelalgia: burning pain, warmth, inflammation, and erythema of the extremities.

Treatment recommendations

As previously established, individuals with PEM often experience burning pain and flushing of the extremities, which can significantly impact their quality of life. According to experts, prevention of flares of PEM should be the primary effort in treatment, relying heavily on quality patient education to reduce the need for pharmacologic and procedural interventions [13,29,30]. Patients are often advised to use safe cooling measures such as time-limited immersion in cool water, use of fans, and elevation of the affected limb. Since exercise is often a common precipitating factor, patients should be encouraged to exercise in well-air-conditioned environments or make activity substitutions such as swimming instead of running [30]. According to the therapeutic ladder proposed by the *Journal of the American Academy of Dermatology*, if a patient fails to achieve a satisfactory level of remission in flares with behavior modification, the next step involves pharmacological interventions, beginning with topical treatments, such as lidocaine patches, being the least invasive [29]. Despite the historical record of erythromelalgia dating as far back as 1832, current treatment options are still primarily based on anecdotal reports of trial and error and case studies rather than robust clinical trials. While modern treatment choices are more informed than in the past, the rarity of PEM and the scarcity of high-quality studies persist as significant roadblocks to establishing treatment guidelines and consistently effective medications.

As noted, genetic investigations into the origin of erythromelalgia pinpointed the NaV1.7 sodium channel as a crucial factor in the condition's pathophysiology, driving the development of novel drugs that target this channel. These advancements in sodium channel understanding have opened the door to innovative therapeutic strategies. Specifically, two promising drugs, funapide (XEN402) and vixotrigine (BIIB074), both selective blockers of NaV1.7, show potential in managing neuropathic pain in patients with PEM [31,32]. These emerging medications signify a significant leap in developing tailored therapies for erythromelalgia, offering renewed prospects for better symptom management and enhanced quality of life for affected individuals. Given the unique variability of symptoms among patients, it is advisable to customize individual treatments based on specific symptomatology. The most effective approach involves a multidisciplinary treatment team consisting of specialists such as dermatologists, neurologists, pain specialists, and psychiatric care providers [13,29].

## Conclusions

This study utilized a literature review and thematic analysis to describe the history and trajectory of knowledge production on PEM. Three periods of scientific development were identified and described. Our findings are significant because they consolidate knowledge on PEM, document the progression of clinical treatment, and bring to light a lesser-known disease. Our findings further confirm what physicians knew starting in the early 19th century - that basic palliative treatments are effective, and they continue to be in our modern time. Current research suggests that it is advisable to customize individual treatments based on specific symptomatology. The most effective approach involves a multidisciplinary treatment team consisting of specialists such as dermatologists, neurologists, pain specialists, and psychiatric care providers.
